# Development of a Predictive Model to Induce Atherogenesis and Hepato-Renal Impairment in Female Rats

**DOI:** 10.3390/biom9110664

**Published:** 2019-10-29

**Authors:** Lucas Pires Guarnier, Paulo Vitor Moreira Romão, Rhanany Alan Calloi Palozi, Aniely Oliveira Silva, Bethânia Rosa Lorençone, Aline Aparecida Macedo Marques, Ariany Carvalho dos Santos, Roosevelt Isaias Carvalho Souza, Karine Delgado Souza, Emerson Luiz Botelho Lourenço, Arquimedes Gasparotto Junior

**Affiliations:** 1 Laboratory of Electrophysiology and Cardiovascular Pharmacology, Faculty of Health Sciences, Federal University of Grande Dourados (UFGD), Dourados, MS 79825-070, Brazil; lucasp.guarnier@gmail.com (L.P.G.); paulovitor_moreiraromao@hotmail.com (P.V.M.R.); palozirhanany@gmail.com (R.A.C.P.); aniiely.oliiveira@gmail.com (A.O.S.); bethaniarosalorencone@hotmail.com (B.R.L.); alinemarques_nutri@hotmail.com (A.A.M.M.); ArianySantos@ufgd.edu.br (A.C.d.S.); RooseveltSouza@ufgd.edu.br (R.I.C.S.); 2 Laboratory of Preclinical Research of Natural Products, Paranaense University (UNIPAR), Umuarama, PR 87502-210, Brazil; karinedsd@hotmail.com (K.D.S.); emerson@prof.unipar.br (E.L.B.L.)

**Keywords:** atherosclerosis, dyslipidemia, hepatic steatosis, kidney failure

## Abstract

Therapeutic approaches for the treatment of dyslipidemia and atherosclerosis have radically changed in recent decades. Part of this advance undeniably stems from basic biomedical research that has provided a better understanding and identification of new therapeutic targets. The aim of this work was to develop a model to induce atherogenesis and hepato-renal impairment in female Wistar rats. The following groups received the respective treatments for 60 days: control animals, non-ovariectomized rats that received an atherogenic diet (NEAD), ovariectomized rats that received an atherogenic diet (NOAD), non-ovariectomized rats that received an atherogenic diet and oral Nω-nitro-l-arginine methyl ester hydrochloride (l-NAME; LEAD), and ovariectomized rats that received an atherogenic diet and oral l-NAME (LOAD). Animals in the NEAD, NOAD, LEAD, and LOAD groups also received methimazole and cholecalciferol daily. Urinary, biochemical, hemodynamic, and electrocardiographic parameters and renal function were assessed. Samples of the liver, heart, kidney, and arteries were collected to investigate redox status and perform histopathological analyses. All of the groups developed dyslipidemia and hepatic steatosis. Only the NEAD group developed arterial lesions that were compatible with fatty streaks. Renal function was significantly impaired in the LEAD and NOAD groups. These results indicate a viable alternative to induce atherogenesis and hepato-renal impairment in female rats.

## 1. Introduction

Atherosclerosis is a multifactorial condition that is associated with chronic inflammatory lesions in the arterial wall. One of the main triggers of atherosclerosis is dyslipidemia, but a wide range of extra-dietary factors may also be involved, including hypertension, smoking, and a sedentary lifestyle [1,2,3]. One of the main factors that is responsible for the genesis of this pathology is low-density lipoprotein (LDL). The deposition of these molecules in the subendothelial space, followed by their oxidation (oxLDL), can be considered a limiting step. Oxidized-LDL particles induce an inflammatory response that is mediated by interleukin (IL)-1 and IL-6, resulting in the activation of adhesion molecules (intercellular adhesion molecule 1 (ICAM-1), vascular adhesion molecule 1 (VCAM-1)) [4]. Thus, monocyte infiltration into the subendothelial space elicits macrophages that phagocytize oxLDL molecules and become foam cells. The inflammatory response is also responsible for the migration of smooth muscle cells that secrete extracellular matrix proteins [5]. More advanced plaques are characterized by a necrotic center and neovascularization, covered by a thin fibrous cap that separates the plaques from the bloodstream. Plaques may remain stable for years but can quickly become unstable, disrupting and triggering thrombus formation and leading to a higher risk of acute cardiovascular events [6]. Consequently, atherosclerosis may lead to renal impairment, coronary heart disease, and stroke [1,7].

In addition to vascular injury itself, dyslipidemias are also involved in a wide range of diseases. Several other organs may be affected by lipid imbalance, among which the heart, kidneys, and liver are notable. The reduction of renal and hepatic function is an important factor that exacerbates subjacent cardiovascular disease and increases its morbidity and mortality [8]. Moreover, steatosis is the main cause of non-alcoholic fatty liver disease and is also associated with higher long-term mortality [3].

Therapeutic approaches for the treatment of dyslipidemia and atherosclerosis have changed radically in recent decades, mainly through a better understanding of the pathophysiological basis of the disease [9]. This advance has undeniably stemmed from basic biomedical research that has provided a better understanding and identification of new therapeutic targets [10]. One of the main problems of existing animal models, however, is their limited ability to reproduce many important pathophysiological aspects of the disease. Some models are promising and easily reproducible, but others have no resemblance to disease progression in humans [11]. Well-established animal models are generally based on the formation of atheromatous plaques that are induced by high-cholesterol diets and genetic manipulation of the pathways that are involved in cholesterol transport and metabolism. The most commonly used animal species for research on atherosclerosis and comorbid pathologies are guinea pigs, hamsters, rabbits, rats, and mice [12]. However, rodents generally have high resistance to the development of atherosclerotic plaques. Murine genetic models are currently the most widely used in preclinical research because they may exhibit different stages of the disease through the activation or inhibition of specific cells or biological pathways. The most used genetic models for studying atherosclerosis are apolipoprotein E homozygous knockout (apoE^−/−^) and LDL receptor homozygous knockout (LDLR^−/−^) models [13]. Despite the speed at which these models develop the disease, they do not accurately reproduce pathophysiological conditions that determine evolution of the disease in humans. Thus, there is currently an important gap in the development of non-genetic rodent models to study the disease. Associations between multiple risk factors (e.g., diet, gender, environmental factors, pharmacological manipulations, and associated pathological conditions) appear to be a plausible alternative to mimic the prevalent conditions that are observed in humans [14]. Therefore, the main objective of the present work was to develop a predictive model to induce atherogenesis and hepato-renal impairment in female rats. We also evaluated the impact of estradiol and nitric oxide (NO) in the proposed models.

## 2. Materials and Methods

### 2.1. Drugs

The following drugs, salts, and solutions were used: ketamine hydrochloride (Syntec, São Paulo, SP, Brazil), xylazine hydrochloride (Syntec, São Paulo, SP, Brazil), and heparin (Hipolabor, Belo Horizonte, MG, Brazil). Nω-nitro-l-arginine methyl ester hydrochloride (l-NAME), phenylephrine (Phe), sodium nitroprusside, acetylcholine, NaCl, KCl, CaCl_2_, MgSO_4_, NaHCO_3_, KH_2_PO_4_, dextrose, ethylenediaminetetraacetic acid, cholesterol, cholecalciferol, colic acid, and methimazole were purchased from Sigma-Aldrich (St. Louis, MO, USA). All of the other reagents were obtained in analytical grade.

### 2.2. Animals and Experimental Design

#### 2.2.1. Animals

Twelve week old female Wistar rats, weighing 220–250 g, were housed in plastic cages with environmental enrichment at 22 ± 2 °C and 55% ± 10% humidity under a 12 h/12 h light/dark cycle. The animals had ad libitum access to food and water. All of the experimental procedures were approved by the Institutional Ethics Committee of Federal University of Grande Dourados (protocol no. 13/2018; approved 22 March 2018) and conducted in agreement with the Brazilian Legal Framework on the Scientific Use of Animals.

#### 2.2.2. Ovariectomy

Female rats were intraperitoneally anesthetized with 100 mg/kg ketamine plus 20 mg/kg xylazine. The animals then underwent laparotomy, and the two ovaries were gently removed. The non-ovariectomized groups (including the control group) underwent laparotomy, but the ovaries were not removed. After the surgical procedure, the animals were hydrated with saline solution (2 mL, subcutaneously, single administration) and received the anti-inflammatory drug ketoprofen (5 mg/kg, orally, every 12 h for 3 days) and antibiotic enrofloxacin (10 mg/kg, subcutaneously, single dose).

#### 2.2.3. Groups and Induction of Atherogenesis and Hepato-Renal Impairment

The animals were randomly allocated to the following groups (*n* = 8/group): normotensive and non-ovariectomized rats that received an standard diet (control rats), normotensive and non-ovariectomized rats that received an atherogenic diet (NEAD), normotensive and ovariectomized rats that received an atherogenic diet (NOAD), normotensive and non-ovariectomized rats that received an atherogenic diet and oral l-NAME (25 mg/kg; LEAD), and normotensive and ovariectomized rats that received an atherogenic diet and oral l-NAME (25 mg/kg; LOAD). The atherogenic diet had the following composition: 64.4% standard diet, 0.5% cholesterol, 0.1% sodium cholate, 5% sucrose, 5% lard, 5% hydrogenated fat, and 20% egg powder. Animals in the NEAD, NOAD, LEAD, and LOAD groups received oral methimazole (2 mg/kg) daily and four intraperitoneal injections (every 2 days) of 175,000 IU/kg cholecalciferol at each dose [15]. All of the treatments were performed for 60 days. Body weight gain was monitored throughout the experimental period.

### 2.3. Experimental Procedures

#### 2.3.1. Renal Function Assay

These experiments were performed on the first day after beginning the treatments and at the end of the experiment period (60 days). All of the animals were placed in metabolic cages for 24 h with food and water ad libitum. Twenty-four hour urine volume, pH, and density were measured. An aliquot was collected for electrolyte (sodium, potassium, calcium, chloride, and magnesium) and creatinine determination.

#### 2.3.2. Electrocardiography

At the end of the experiment period, the animals were intramuscularly anesthetized with 100 mg/kg ketamine plus 20 mg/kg xylazine. After anesthesia, all of the rats underwent electrocardiography (ECG) according to the methodology of Romão et al. [16]. Using four alligator clips, the electrodes were positioned on the animal’s two forelimbs and two hindlimbs. An acclimatization period of 5 min elapsed, and ECG waves were recorded for 5 min. Electrocardiography was recorded using a 12-lead ECG recorder (WinCardio, Micromed, Brasilia, Brazil) [16].

#### 2.3.3. Blood Pressure Measurement

After ECG recording and while under deep anesthesia, systolic blood pressure (SBP) and mean arterial pressure (MAP) were determined by the direct method according to Gasparotto Junior et al. [17]. A subcutaneous injection of heparin (30 IU) was first administered. The left carotid artery was then isolated, cannulated, and connected to a pressure transducer that was coupled to a PowerLab system with Chart 7.1 software (ADInstruments, Castle Hill, Australia), and SBP, DBP (diastolic blood pressure), and MAP were recorded for 20 min.

#### 2.3.4. Renal and Mesenteric Vascular Bed Reactivity

After the direct blood pressure measurements, the mesenteric vascular bed (MVB) and left kidney were isolated and prepared for perfusion according to previously described methods [18]. The renal artery was first cannulated, followed by cannulation of the superior mesenteric artery. The kidney and MVB were removed, isolated, coupled in a perfusion system, and continuously perfused with physiological saline solution (PSS; 119 mM NaCl, 4.7 mM KCl, 2.4 mM CaCl_2_, 1.2 mM MgSO_4_, 25.0 mM NaHCO_3_, 1.2 mM KH_2_PO_4_, 11.1 mM dextrose, and 0.03 mM ethylenediaminetetraacetic acid) that was aerated with 95% O_2_ and 5% CO_2_ at a constant flow rate of 4 mL/min. Changes in perfusion pressure (PP; mmHg) were captured by a pressure transducer that was connected to the PowerLab system and Chart 7.1 software (ADInstruments, Castle Hill, Australia). After a stabilization time of 30 min, tissue integrity was assessed with a bolus injection of KCl (120 mmol). Different doses of Phe were then administered in the MVB (1, 3, 10, and 30 nmol, 10–30 µL) and kidney (0.1, 0.3, 1, and 3 nmol, 10–30 µL) preparations. Angiotensin II (AngII) was administered only in the kidney preparations (1, 3, 10, and 30 pmol, 10–30 µL). After a new stabilization time of 30 min, the tissues were continuously perfused with PSS plus 3 µM Phe to induce a prolonged increase in perfusion pressure. After stabilization of the contractile process, vascular reactivity to acetylcholine (Ach; MVB: 1, 3, 10, and 30 pmol; kidneys: 0.03, 0.1, 0.3, and 1 nmol) and sodium nitroprusside (SNP; MVB: 0.1, 0.3, 1, and 3 pmol; kidneys: 0.3, 1, 3, and 10 nmol) was evaluated. A 10 min period elapsed between each drug administration. At the end of the experiments, the animals were euthanized by deep anesthesia in a saturation chamber (isoflurane with >20% saturation).

#### 2.3.5. Biochemical Analyses

Immediately before euthanasia, blood samples were collected from the previously cannulated left carotid artery. Serum was obtained by centrifugation at 1500× *g* for 10 min. Alanine aminotransferase (ALT), aspartate aminotransferase (AST), indirect bilirubin, direct bilirubin, total bilirubin, total protein, albumin, globulin, the albumin/globulin ratio, creatinine, urea, triglycerides (TG), total cholesterol (TC), high-density lipoprotein cholesterol (HDL-C), alkaline phosphatase, γ-glutamyl transpeptidase (GGT), sodium, calcium, magnesium, chloride, and potassium levels were measured using an automated biochemical analyzer (Roche Cobas Integra 400 plus). Low-density lipoprotein cholesterol (LDL-C) and very-low-density lipoprotein cholesterol (VLDL-C) were calculated according to the Friedwald formula [5]. Serum oxLDL, nitrotyrosine (NT), soluble VCAM-1 (sVCAM-1), soluble ICAM-1 (sICAM-1), IL-1β, and IL-6 levels were measured using an enzyme-linked immunosorbent assay (ELISA; BD Biosciences, San Jose, CA, USA). Malondialdehyde (MDA) levels were measured using an MDA assay kit (Cayman Chemical, Ann Arbor, MI, USA). Estradiol, thyroid-stimulating hormone (TSH), and free triiodothyronine (T3) were measured using a chemiluminescence immunoassay. Hepatic triglyceride content was measured using a previously described method [19].

#### 2.3.6. Relative Organ Weight

After euthanasia, the heart, right kidney, and liver were cleaned, weighed, and longitudinally sectioned. The relative organ weight was determined as the following: Relative Weight (RW)% = absolute organ weight × 100/body weight.

#### 2.3.7. Tissue Redox Status

Samples of the aorta, heart (ventricular muscle), and right kidney were homogenized in phosphate buffer (pH 6.5) and centrifuged at 5269× *g* for 20 min at 4 °C. Different dilutions were used for each tissue. The following enzymes were evaluated in the supernatant: catalase (CAT; according to [20]) and superoxide dismutase (SOD; according to [21]). Lipoperoxidation (LPO) rate was measured using the FOX2 method adapted by Jiang et al. [22]. The results were expressed as the amount of protein in the homogenates. The protein concentration of the samples was determined using the Bradford method [23].

#### 2.3.8. Histopathology and Morphometry

Parts of the left ventricle, right kidney, liver, subclavian artery, and right carotid artery were placed in 10% buffered formalin. The samples were then dehydrated in alcohol, cleared with xylene, and embedded in paraffin. The samples were sectioned (5 mm) and stained with hematoxylin-eosin and orcein (arteries) and examined under a light microscope. Right and left ventricles and interventricular septum were measured. The intima layer of the subclavian and carotid artery was also measured. We assembled several blades with the entire arterial branch. All neointimal formations were identified per slide. For each atherosclerotic formation, six lines of division were established. For each neointimal formation, we performed six measurements, and one mean for each slide was calculated. When all of the slides were evaluated, a new mean for each animal was calculated. The area of the microscope was adjusted using an ocular micrometer with a 100× objective to determine the size of the field per square micrometer (µm²). All images were obtained and evaluated using Motic Images Plus 2.0 software.

### 2.4. Statistical Analyses

Statistical analyses were performed using analysis of variance (ANOVA) followed by Bonferroni’s post hoc test. The results are expressed as the mean ± standard error of the mean (SEM) of *n* = 8 animals per group. Values of *p* < 0.05 were considered statistically significant.

## 3. Results

### 3.1. Renal Function

Different urinary parameters in the various groups over 60 days of treatment are presented in Table 1. Animals in the LEAD and LOAD groups presented significantly lower urinary chloride, potassium, and sodium levels compared with the control group. The LOAD group exhibited significantly lower urinary creatinine levels than the control group.

### 3.2. Electrocardiographic Parameters

No significant electrocardiographic changes were observed among the different experimental groups (Table 2).

### 3.3. Blood Pressure and Heart Rate Values

Blood pressure and heart rate data in the different groups are presented in Table 3. We found a significant increase in SBP only in the LOAD group when compared with the control.

### 3.4. Biochemical Analyses

Biochemical parameters in the various experimental groups over 60 days of treatment are presented in Table 4. The LEAD and LOAD groups exhibited a significant increase in serum globulin levels and the albumin/globulin ratio compared with the control. Serum TG, TC, LDL-C, VLDL-C, and HDL-C levels significantly increased in the NEAD, NOAD, LEAD, and LOAD groups. HDL-C levels were significantly higher in the NOAD, LEAD, and LOAD compared with the NEAD group. LDL-C levels were significantly higher in the NEAD group when compared with all of the other experimental groups. Hepatic TG values were also significantly higher in the NEAD, NOAD, LEAD, and LOAD groups compared with the control.

Oxidized LDL, MDA, and NT levels were significantly elevated in all of the experimental groups compared with control-rats. The NEAD, LEAD, and LOAD groups exhibited significant increases in sVCAM-1, sICAM-1, IL-6, and IL-1β levels compared with the control. As expected, estradiol levels were significantly lower in ovariectomized animals.

### 3.5. Renal and Mesenteric Vascular Bed Reactivity

Phe, ACh, and SNP reactivity in the isolated and perfused MVB in the various groups are presented in Table 5. The LEAD and LOAD groups exhibited higher Phe reactivity at 10 nmol compared with the control group. Moreover, at 30 nmol, the NOAD, LEAD, and LOAD groups exhibited a significant increase in Phe reactivity compared with the NEAD and control groups. Acetylcholine reactivity significantly decreased in the LOAD group at all doses tested compared with the NEAD, NOAD, LEAD, and control groups. Similarly, the LEAD group exhibited a significant decrease in ACh reactivity at 10 pmol.

Renal vascular reactivity in the various groups over 60 days of treatment is presented in Table 6. Isolated kidney preparations in the LOAD group exhibited a significant decrease in Phe reactivity. At 1 nmol, Phe reactivity was significantly lower in the LOAD group than in the control, NEAD, NOAD, and LEAD groups. Similarly, preparations that were obtained from animals in the LOAD group exhibited a significant reduction of the constricting effects of Ang II (1 pmol) compared with the control, NEAD, NOAD, and LEAD groups. All of the experimental groups exhibited a significant reduction of the vasodilatory effects of ACh (0.1 nmol) compared with the control group.

### 3.6. Body Weight and Relative Weight of Heart, Kidney, and Liver

The initial and final body weight and relative weight of the heart and right kidney did not show any statistically significant changes compared to the different experimental groups (Table 7). The relative weight of the liver in the NEAD, NOAD, LEAD, and LOAD groups were significantly increased when compared with the control group (Table 7).

### 3.7. Tissue Redox Status

The levels of SOD, CAT, and LPO in cardiac, renal, and arterial tissues are presented in Table 8. In ventricular muscle, we found a significant increase in SOD and CAT levels in the LOAD group. Similarly, in kidney tissue, the LOAD and NEAD groups exhibited a significant increase in SOD and LPO levels. The LEAD group exhibited only an increase in LPO levels. The NOAD group exhibited a significant reduction of CAT levels in renal tissue. In arterial tissue, the LOAD group exhibited a significant increase in SOD, CAT, and LPO levels compared with the control group. Similarly, the NEAD and LEAD groups exhibited significant increases in CAT levels. In arterial tissue, LPO levels were significantly lower in the NOAD group than in the NEAD, LEAD, LOAD, and control groups.

### 3.8. Cardiac and Arterial Morphometry

The interventricular septum and intimal layer of the subclavian and carotid arteries in the NEAD group exhibited significant thickening compared with all of the other experimental groups (Table 9). The right and left ventricles exhibited no significant changes in any of the groups.

### 3.9. Histopathological Analysis

**Figure 1:**
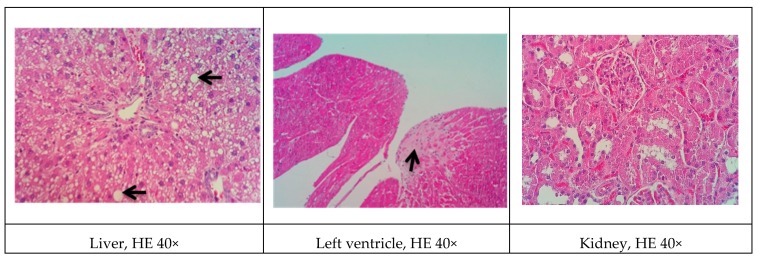

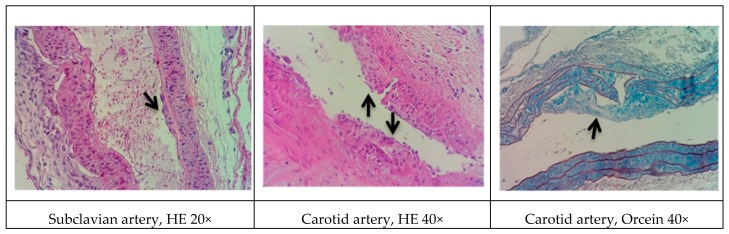
Representative cross-sections of the liver, left ventricle, kidney, and subclavian and carotid arteries in the NEAD group. Black arrows indicate cardiac fibrosis, lipid macrovesicles in the liver, and lipid striae in the arteries. HE, hematoxylin-eosin staining; NEAD, normotensive and non-ovariectomized rats that received the atherogenic diet, methimazole, and cholecalciferol.

**Figure 2:**
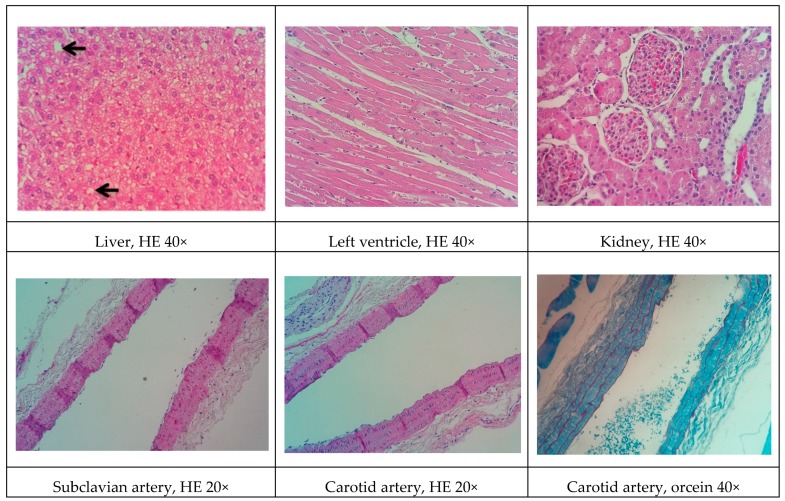
Representative cross-sections of the liver, left ventricle, kidney, and subclavian and carotid arteries in the NOAD group. Black arrows indicate lipid macrovesicles in the liver. HE, hematoxylin-eosin staining; NOAD, normotensive and ovariectomized rats that received the atherogenic diet, methimazole, and cholecalciferol.

**Figure 3:**
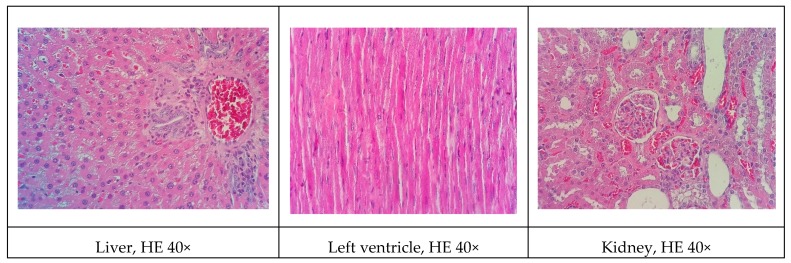

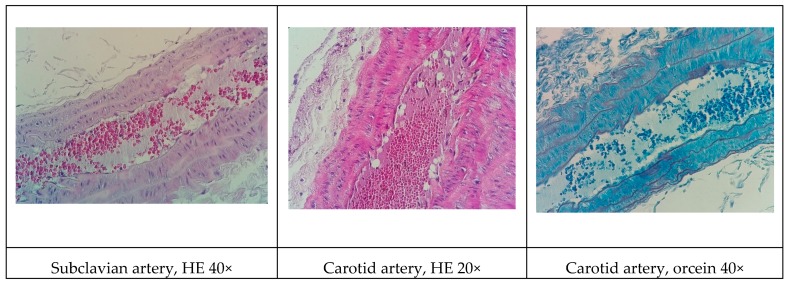
Representative cross-sections of the liver, left ventricle, kidney, and subclavian and carotid arteries in the LEAD group. HE, hematoxylin-eosin staining; LEAD, normotensive and non-ovariectomized rats that received the atherogenic diet, methimazole, cholecalciferol, and l-NAME.

**Figure 4:**
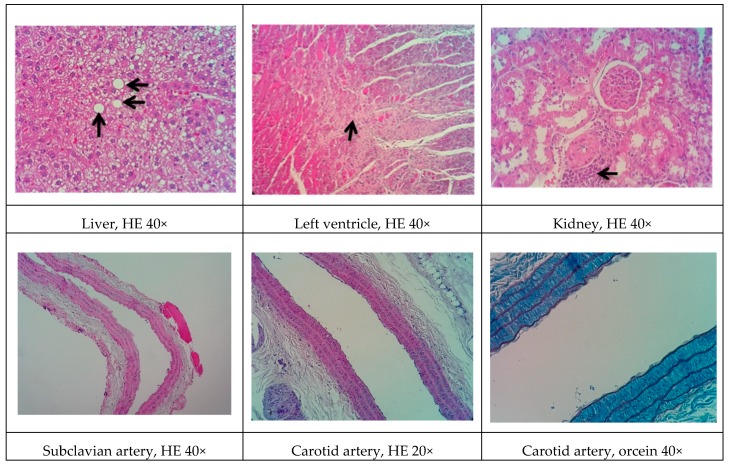
Representative cross-sections of the liver, left ventricle, kidney, and subclavian and carotid arteries in the LOAD group. Black arrows indicate cardiac fibrosis, lipid macrovesicles in the liver, and inflammatory infiltrate in renal tissue. HE, hematoxylin-eosin staining; LOAD, normotensive and ovariectomized rats that received the atherogenic diet, methimazole, cholecalciferol, and l-NAME.

**Figure 5:**
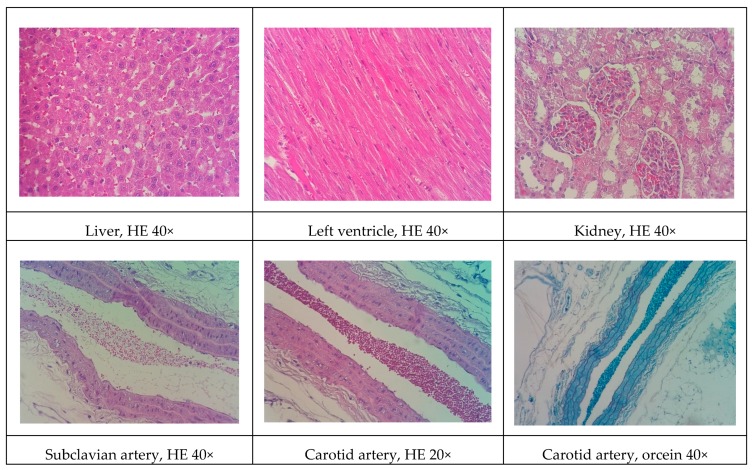
Representative cross-sections of the liver, left ventricle, kidney, and subclavian and carotid arteries in the control group. HE, hematoxylin-eosin staining.

Representative histological images of the liver, left ventricle, kidney, and arteries (subclavian and carotid) in the various experimental groups are shown in Figure 1, Figure 2, Figure 3, Figure 4 and Figure 5. All of the animals that received the high-fat diet exhibited significant lipid accumulation in periportal and mediozonal hepatocytes, with a discrete-to-moderate focus of mononuclear inflammatory infiltrate. The NEAD (Figure 1) and LOAD (Figure 4) groups exhibited the diffuse formation of cytoplasmic lipid macro- and microvesicles and slight random individual hepatocyte necrosis. In the NOAD (Figure 2) and LEAD (Figure 3) groups, hepatic steatosis acquired a microvesicular aspect without displacing the cell nucleus. In cardiac tissue in the NEAD (Figure 1) and LOAD (Figure 4) groups, we observed a focally extensive area of fibrous tissue deposition that was associated with mild cardiac fiber necrosis and mild mononuclear inflammatory infiltrates into the left ventricular myocardium near the endocardium. In the kidney in the LOAD group, we found some areas of mononuclear inflammatory infiltrate and the presence of eosinophilic amorphous material (Figure 4). Only the NEAD group exhibited atheromatous lesions (Figure 1).

## 4. Discussion

Animal models cannot fully reproduce all stages of the development of atherosclerosis in humans. However, in many cases, they can predict important pathophysiological mechanisms of the disease and assist in the identification of new therapeutic targets [14]. Non-genetic rodent models (unlike rabbits) are unable to form short-term atherosclerotic plaques with a high-fat diet (HFD). Therefore, most rodent models consist of a combination of high cholesterol or a HFD and the administration of suprapharmacological doses of vitamin D3 or antithyroid drugs [24]. Several studies to date have utilized mixed models with a notable lack of consensus on precise dietary ingredients and exposure times (e.g., 6–17 weeks). Additionally, no consensus has been reached with regard to animal age, cholesterol or fat content, pharmacological adjuvants, and the presence or absence of sex hormones and hypertension, among other factors [24]. Given these discrepant data, we performed a 60 day treatment regimen that was based on two assumptions. First, when using rats in different models of heart disease, functional changes occur rapidly (within 1 or 2 weeks), but structural changes tend to appear later (e.g., after 6–8 weeks) [25]. Second, when using other non-rodent species, such as rabbits, significant results have been obtained after 60 days of dietary exposure [26].

Despite the fact that females are known to have specific cardiovascular characteristics, the treatments that are specifically directed toward this gender are nonexistent [27]. Additionally, there is a dearth of animal models of atherosclerotic disease for this genus. Estradiol has antioxidant and cardioprotective effects, but the scientific community has a tendency to test only male animals [28]. In fact, a recent review of non-genetic rat models for atherosclerosis research reported only one study that was conducted with females [24]. Thus, we used the same HFD associated with methimazole and cholecalciferol in the four experimental groups (NEAD, NOAD, LEAD, and LOAD). However, in two groups (NOAD and LOAD), we removed the ovaries to mimic the clinical condition that occurs after menopause in women. In one group with ovaries (LEAD) and another group without ovaries (LOAD), we also administered the NO synthase inhibitor l-NAME to reduce the body supply of NO, an important vasodilator and antioxidant and antiplatelet agent [29]. No studies of which we are aware have used l-NAME in the proposed model. We included l-NAME to increase LDL oxidation, a limiting step in atheromatous plaque formation. Additionally, NO is an important endothelial mediator that controls vascular tone, and NO inhibition can induce endothelial dysfunction that precedes atherosclerotic lesions [30].

In rodents, a standard HFD alone is incapable of inducing more advanced stages of atherosclerosis. Data have shown that an HFD leads only to earlier events of the disease, such as slight thickening of the intimal layer of the arteries [31]. Thus, we expanded the diet to reduce total purified cholesterol and included 20% egg powder, offering a higher content of protein and other fat sources. We also added sucrose and hydrogenated fat. Several studies indicate that diets that are rich in sucrose or hydrogenated fat may induce endothelial dysfunction, raise blood pressure, and cause long-term cardiovascular and renal complications [32,33]. In addition to diet, one alternative to reduce rodent resistance to atherosclerosis is the co-administration of drugs that favor development of the disease. Studies have shown that the co-administration of cholecalciferol facilitates the induction of significant vascular and soft tissue calcifications [34]. Another synergistic effect can be obtained with a drug that reduces metabolism in animals. The use of antithyroid drugs (e.g., methimazole and propylthiouracil) has shown promising results. Fu et al. [35] reported that male Sprague-Dawley rats that were treated with cholecalciferol (600,000 IU/kg) combined with a HFD (81.3% basic feed, 10% lard, 3% cholesterol, 0.5% sodium cholate, and 5% sugar) and 0.2% propylthiouracil (for 17 weeks) developed dyslipidemia and lipid striae in different arterial branches. Additionally, signs of atherogenesis were observed in rats with a shorter exposure time (10 weeks) after treatment with cholecalciferol (70 U/kg, continuous administration for 3 days), propylthiouracil (0.7%), and a HFD (80.3% normal diet, 11% fat, 4.5% cholesterol, 1.5% sodium cholate, and 4% refined sugar) [36].

Although all the models proposed in this study have induced relevant pathophysiological changes, including dyslipidemia and steatosis, one fact caught our attention. Only the NEAD group showed increased LDL-C and significant atherosclerotic lesions. Several data have shown that estrogen promotes natural protection to the endothelium and is related to the decrease of serum cholesterol, especially LDL-C. This natural protection occurs through the regulation of anti-atherogenic agents, such as nitric oxide and indirect effects on the liver [37]. In humans, estrogens lower circulating LDL-C and increase HDL-C [38]. On the other hand, it is currently known that estrogen has been shown to lower hepatic and plasma proprotein convertase subtilisin/kexin type 9 (PCSK9) levels in animals and humans. Changes in circulating PCSK9 contribute to the established increase in LDL-C [39]. In fact, PCSK9 regulates LDL-C by promoting the endosomal/lysosomal degradation of the LDL-C receptor (LDLR) [40]. We think that the probable cause of the contradictory results may be associated with the hepatic alterations induced by an HFD. Hepatic steatosis modulates lipid regulatory genes, including PCSK9 and the LDLR, contributing to increasing LDL-C levels and atherosclerosis [41].

Another significant difference among the present experimental models involved cardiovascular hemodynamics. Interestingly, l-NAME raised blood pressure only in the LOAD group. This is likely attributable to the fact that non-ovariectomized females (LEAD group) had high levels of circulating estradiol, which likely contributed to the observed cardioprotective effects [28,42]. As expected, we observed higher Phe reactivity and lower ACh reactivity in the mesenteric vessels in the l-NAME-treated groups, with even more pronounced effects in ovariectomized animals (LOAD group). This was mainly attributable to the antioxidant effects of estradiol, which exerts protective effects on endothelial function even in the absence of NO [43]. Although these results show an increase in cardiovascular risk factors in the LOAD group, these changes do not specifically reflect atherosclerotic lesions. The effects on local oxidative stress and LDL-C and HDL-C levels may play a more important role in this case.

Some of the data on cardiac architecture were also interesting. Although we did not detect any significant electrocardiographic alterations, we also observed important cardiac structural changes, including fibrosis and hypertrophy, especially in the NOAD group. Hypertension is a well-known risk factor for the development of ventricular hypertrophy. In the present study, only the LOAD group exhibited a significant increase in SBP. Nevertheless, no trace of cardiac muscle hypertrophy was found in this group. We believe this was attributable to the slight elevation of blood pressure and the time of exposure to these hemodynamic changes [44]. It is unclear whether cholesterol alone is sufficient in causing cardiac hypertrophy. Several studies have shown that cholesterol loading significantly increases the cellular surface area and upregulates the hypertrophy marker gene β-myosin heavy chain. Cholesterol loading alone activates the extracellular signal-regulated kinase (ERK)/mitogen-activated protein kinase (MAPK) and phosphatidylinositol-3-kinase (PI3K)/AKT pathways. Conversely, cholesterol-induced hypertrophic features, such as an increase in cell surface area and the expression of β-myosin heavy chain mRNA, are markedly inhibited by the PI3K kinase inhibitor LY294002. Therefore, cholesterol may play a key role in the development of cardiac hypertrophy through activation of the PI3K/AKT pathway [45].

In general, renal function in the experimental groups was significantly influenced by l-NAME administration. The administration of l-NAME in the proposed models did not improve the atherogenic profile and actually aggravated renal function. Concentric inflammatory areas in the kidneys were commonly observed among the histological findings in these animals. l-NAME may reduce renal function by causing less dilation of the efferent arteriole and reducing glomerular perfusion pressure and filtration rate [46]. Thus, in the proposed model, the addition of l-NAME had a greater impact on renal function than on the development of the atherosclerotic process.

The present study had two important limitations. First, during execution of the protocols, we could not determine why only the NEAD group exhibited higher LDL levels and lower HDL levels if the diet that was offered to the animals was the same. The presence of estradiol and absence of l-NAME may be a possible reason, but further studies are needed to examine this possibility. Second, the use of more effective methodologies to raise blood pressure could have been employed. The use of spontaneously hypertensive rats or animals with renovascular hypertensive may be promising, which would simulate conditions that are closer to the human condition. Nevertheless, the present findings reveal a new non-genetic rat model of atherosclerotic disease that may contribute to further research on the prevention, staging, and regression of atherosclerosis and its comorbidities.

## 5. Conclusions

The present study identified a viable alternative to induce atherogenesis and hepato-renal impairment in female rats. Although the absence of estradiol and inhibition of NO synthesis did not directly affect hepatic changes or atherosclerotic plaque formation, renal function appeared to be highly dependent on NO levels.

## Figures and Tables

**Table 1:** Urinary parameters in the various groups over 60 days of treatment.

Parameter	NEAD	NOAD	LEAD	LOAD	Control
Urinary volume (mL/100 g/24 h)	12.54 ± 4.70	16.04 ± 5.35	19.42 ± 8.19	15.21 ± 1.58	12.6 ± 2.30
Chloride (mmol/dL)	171.5 ± 12.92	136.79 ± 22.05	82.37 ± 11.25 ^a^	73.50 ± 11.81 ^a^	141.07 ± 18.31
Magnesium (mg/dL)	20.45 ± 8.56	32.53 ± 7.81	19.465 ± 8.78	21.26 ± 10.70	14.57 ± 4.04
Potassium (mmol/L)	112.20 ± 19.62	124.42 ± 31.72	70.93 ± 28.54 ^a^	65.17 ± 17.91 ^a^	140.16 ± 14.25
Sodium (mEq/L)	108.68 ± 29.89	127.72 ± 126.98	75.62 ± 15.33 ^a^	63.08 ± 16.45 ^a^	120.32 ± 13.13
Calcium (mg/dL)	45.47 ± 10.31	54.26 ± 21.53	32.85 ± 9.34	47.32 ± 19.83	26.72 ± 8.10
Creatinine (mg/dL)	40.71 ± 24.74	49.74 ± 21.17	30.38 ± 11.34	28.03 ± 6.43 ^a^	54.47 ± 6.30
pH	6.16 ± 0.48	6.03 ± 0.56	6.33 ± 0.10	6.03 ± 0.20	7.1 ± 0.43

The data are expressed as the mean ± SEM of *n* = 8 rats per group compared with the control group (^a^ *p* < 0.05) using one-way ANOVA followed by Bonferroni’s post hoc test. LEAD, normotensive and non-ovariectomized rats that received the atherogenic diet, methimazole, cholecalciferol, and oral Nω-nitro-l-arginine methyl ester hydrochloride (l-NAME); LOAD, normotensive and ovariectomized rats that received the atherogenic diet, methimazole, cholecalciferol, and l-NAME; NEAD, normotensive and non-ovariectomized rats that received the atherogenic diet, methimazole, and cholecalciferol; NOAD, normotensive and ovariectomized rats that received the atherogenic diet, methimazole, and cholecalciferol.

**Table 2:** Electrocardiographic parameters in the various groups over 60 days of treatment.

Parameter	NEAD	NOAD	LEAD	LOAD	Control
PR segment (ms)	45.29 ± 1.59	49 ± 3.86	45.29 ± 3.61	42.5 ± 2.90	46.67 ± 2.72
QRS segment (ms)	39.57 ± 1.67	36 ± 0.95	38.71 ± 1.80	37.8 ± 1.06	37.89 ± 1.36
QT segment (ms)	131.6 ± 9.41	141 ± 9.41	141.7 ± 10.61	132.4 ± 11.17	134.1 ± 7.66
QTC segment (ms)	234.9 ± 15.69	256.1 ± 12.92	261.3 ± 25.07	242 ± 18.44	232.7 ± 9.891
P wave (mV)	0.063 ± 0.003	0.083 ± 0.011	0.084 ± 0.01	0.089 ± 0.006	0.084 ± 0.004
Q wave (mV)	−0.013 ± 0.002	−0.011 ± 0.001	−0.011 ± 0.001	−0.012 ± 0.001	−0.011 ± 0.002
R wave (mV)	0.38 ± 0.02	0.43 ± 0.04	0.38 ± 0.04	0.37 ± 0.02	0.37 ± 0.01

The data are expressed as the mean ± SEM of *n* = 8 rats per group. Statistical analyses were performed using one-way ANOVA followed by Bonferroni’s post hoc test. LEAD, normotensive and non-ovariectomized rats that received the atherogenic diet, methimazole, cholecalciferol, and l-NAME; LOAD, normotensive and ovariectomized rats that received the atherogenic diet, methimazole, cholecalciferol, and L-NAME; NEAD, normotensive and non-ovariectomized rats that received the atherogenic diet, methimazole, and cholecalciferol; NOAD, normotensive and ovariectomized rats that received the atherogenic diet, methimazole, and cholecalciferol. mV, millivolt; ms, millisecond.

**Table 3:** Blood pressure and heart rate values in the various groups over 60 days of treatment.

Parameter	NEAD	NOAD	LEAD	LOAD	Control
DBP (mmHg)	63.24 ± 4.26	60.42 ± 5.12	60.25 ± 4.32	58.82 ± 3.31	61.88 ± 3.84
SBP (mmHg)	100.55 ± 9.05	100.74 ± 10.88	109.24 ± 6.77	117.3 ± 3.88 ^a^	99.7 ± 4.43
MAP (mmHg)	84.95 ± 6.33	69.26 ± 3.98	83.33 ± 4.64	75.86 ± 3.51	68.84 ± 6.24
HR (bpm)	214.9 ± 19.16	211.6 ± 16.9	159.5 ± 11.42	206.8 ± 15.35	175.3 ± 7.319

The data are expressed as mean ± SEM of *n* = 8 rats per group compared with the control group (^a^ *p* < 0.05) using one-way ANOVA followed by Bonferroni’s post hoc test. DBP, diastolic blood pressure; HR, heart rate. LEAD, normotensive and non-ovariectomized rats that received the atherogenic diet, methimazole, cholecalciferol, and l-NAME; LOAD, normotensive and ovariectomized rats that received the atherogenic diet, methimazole, cholecalciferol, and l-NAME; MAP, mean arterial pressure; NEAD, normotensive and non-ovariectomized rats that received the atherogenic diet, methimazole, and cholecalciferol; NOAD, normotensive and ovariectomized rats that received the atherogenic diet, methimazole, and cholecalciferol; SBP, systolic blood pressure;

**Table 4:** Biochemical parameters in the various groups over 60 days of treatment.

Parameter	NEAD	NOAD	LEAD	LOAD	Control
Total protein (g/dL)	6.21 ± 0.83	6.51 ± 1.14	6.62 ± 0.48	6.64 ± 0.66	6.24 ± 0.59
Albumin (g/dL)	3.61 ± 0.42	3.85 ± 0.72	3.53 ± 0.45	3.64 ± 0.52	4.10 ± 0.35
Globulin (g/dL)	2.60 ± 0.51	2.65 ± 0.53	3.08 ± 0.26 ^a^	3.01 ± 0.16 ^a^	2.12 ± 0.18
Alb/Glob	1.42 ± 0.28	1.47 ± 0.26	1.15 ± 0.19 ^a^	1.22 ± 0.13 ^a^	1.91 ± 0.13
AST (U/L)	120.71 ± 46.02	127.72 ± 103.50	116.14 ± 52.50	124.12 ± 46.75	171.83 ± 43.21
ALT (U/L)	56.18 ± 14.51	83.42 ± 98.50	57.5 ± 19.98	58.65 ± 10.56	82.77 ± 19.20
GGT (U/L)	1.71 ± 0.75	1.57 ± 0.78	1.45 ± 0.89	0.87 ± 0.69	1.62 ± 0.74
AP (U/L)	114.57 ± 39.13	114.14 ± 49.49	124.6 ± 40.97	113.12 ± 54.65	103.25 ± 65.22
IB (mg/dL)	0.035 ± 0.021	0.031 ± 0.015	0.036 ± 17.98	0.041 ± 0.014	0.033 ± 0.014
DB (mg/dL)	0.013 ± 0.008	0.017 ± 0.019	0.026 ± 0.018	0.016 ± 0.013	0.015 ± 0.009
TB (mg/dL)	0.045 ± 0.021	0.048 ± 0.016	0.062 ± 0.013	0.057 ± 0.023	0.048 ± 0.018
TG (mg/dL)	156.11 ± 10.03 ^a^	177.22 ± 15.12 ^a^	147.22 ± 12.03 ^a^	150.19 ± 11.15 ^a^	69.12 ± 7.11
Hepatic TG (mmol/L)	125.11 ± 5.08 ^a^	123.32 ± 2.52 ^a^	138.11 ± 7.91 ^a^	145.30 ± 7.24 ^a^	95.31 ± 4.73
TC (mg/dL)	168.14 ± 25.33 ^a^	161.57 ± 39.04 ^a^	197 ± 29.02 ^a^	160.25 ± 25.12 ^a^	61.37 ± 14.02
HDL-C (mg/dL)	40.21 ± 8.21 ^a^	81.29 ± 9.32 ^a,d^	92.97 ± 10.11 ^a,d^	80.71 ± 9.21 ^a,d^	27.12 ± 7.04
VLDL-C (mg/dL)	33.63 ± 7.71 ^a^	35.44 ± 4.77 ^a^	29.44 ± 4.01 ^a^	30.04 ± 3.45 ^a^	13.82 ± 3.22
LDL-C (mg/dL)	96.8 ± 9.32 ^a,b,c,e^	45.33 ± 5.12 ^a^	76.11 ± 7.01 ^a^	50.21 ± 4.11 ^a^	20.01 ± 2.33
oxLDL (ng/mL)	0.80 ± 0.10 ^a^	0.92 ± 0.09 ^a^	1.32 ± 0.11 ^a^	2.07 ± 0.13 ^a,b,c,d^	0.24 ± 0.05
NT (μmol/L)	0.020 ± 0.002 ^a^	0.021 ± 0.003 ^a^	0.023 ± 0.003 ^a^	0.025 ± 0.004 ^a^	0.011 ± 0.002
MDA (mmol/L)	3.00 ± 0.11 ^a^	3.13 ± 0.14 ^a^	3.53 ± 0.22 ^a^	4.15 ± 0.33 ^a^	2.13 ± 0.19
sVCAM-1 (ng/L)	3.98 ± 0.12 ^a^	3.13 ± 0.37	3.78 ± 0.17 ^a^	4.00 ± 0.21 ^a^	2.50 ± 0.11
sICAM-1 (ng/L)	8.20 ± 1.16 ^a^	6.16 ± 1.24	8.72 ± 0.94 ^a^	8.70 ± 1.03 ^a^	4.01 ± 0.57
IL-6 (ng/L)	301.55 ± 40.3 ^a^	250.54 ± 45.1	300.11 ± 33.4 ^a^	334.23 ± 22.3 ^a,c^	165.21 ± 22.2
IL-1β (pg/mL)	430.17 ± 30.1 ^a^	390.32 ± 44.2	460.22 ± 30.1 ^a^	521.17 ± 30.1 ^a,c^	333.45 ± 40.3
Creatinine (mg/dL)	0.36 ± 0.06	0.39 ± 0.13	0.42 ± 0.02	0.39 ± 0.076	0.34 ± 0.09
Urea (mg/dL)	47.02 ± 10.09	38.92 ± 6.16	50.22 ± 0.07	45.07 ± 9.34	52.06 ± 11.34
Sodium (mmol/L)	136.28 ± 5.87	135.85 ± 10.76	141.4 ± 5.27	143.37 ± 6.73	142.37 ± 6.04
Calcium (mg/dL)	11.41 ± 0.85	11.2 ± 1.70	11.46± 0.36	11.62 ± 0.85	10.55 ± 0.77
Potassium (mmol/L)	4.95 ± 1.33	4.37 ± 1.01	5.36 ± 0.77	4.27 ± 0.66	4.03 ± 0.39
Magnesium (mg/dL)	2.45 ± 0.42	2.42 ± 1.01	2.48 ± 0.22	2.19 ± 0.12	2.32 ± 0.14
Chloride (mmol/L)	98.14 ± 5.14	97.85 ± 6.36	102.8 ± 4.81	104.87 ± 3.39	103.87 ± 3.87
Estradiol (pg/mL)	45.99 ± 9.43	1.01 ± 0.31 ^a^	55.01 ± 10.02	0.98 ± 0.21 ^a^	40.22 ± 11.21
Free T3 (ng/mL)	0.54 ± 0.14	0.60 ± 0.11	0.51 ± 0.09	0.48 ± 0.08	0.50 ± 0.10
TSH (ng/mL)	5.21 ± 0.54	5.12 ± 0.77	4.93 ± 0.59	5.01 ± 0.92	4.75 ± 0.77

The data are expressed as mean ± SEM of *n* = 8 rats per group compared with the control group (^a^ *p* < 0.05), LEAD group (^b^ *p* < 0.05), NOAD group (^c^ *p* < 0.05), NEAD group (^d^ *p* < 0.05), and LOAD group (^e^ *p* < 0.05) using one-way ANOVA followed by Bonferroni’s post hoc test. ALT, alanine aminotransferase; AP, alkaline phosphatase; AST, aspartate aminotransferase; DB, direct bilirubin; GGT, γ-glutamyltransferase; HDL-C, high-density lipoprotein cholesterol; IB, indirect bilirubin; IL-6, interleukin-6; IL-1β, interleukin-1β; LDL-C, low density lipoprotein cholesterol; LEAD, normotensive and non-ovariectomized rats that received the atherogenic diet, methimazole, cholecalciferol, and L-NAME; LOAD, normotensive and ovariectomized rats that received the atherogenic diet, methimazole, cholecalciferol, and l-NAME; MDA, malondialdehyde; NEAD, normotensive and non-ovariectomized rats that received the atherogenic diet, methimazole, and cholecalciferol; NOAD, normotensive and ovariectomized rats that received the atherogenic diet, methimazole, and cholecalciferol; NT, nitrotyrosine; oxLDL, oxidized low-density lipoprotein; sICAM, soluble intercellular adhesion molecule-1; sVCAM-1, soluble vascular cell adhesion molecule-1; T3, triiodothyronine; TB, total bilirubin; TC, total cholesterol; TG, triglycerides; TSH, thyroid-stimulating hormone; VLDL-C, very-low-density lipoprotein cholesterol.

**Table 5:** Mesenteric vascular bed reactivity in the various groups over 60 days of treatment. The data are presented as perfusion pressure variation (mmHg) after Phe, ACh, and SNP administration.

Phe (nmol)	NEAD	NOAD	LEAD	LOAD	Control
1	1.74 ± 0.26	1.69 ± 0.43	1.51 ± 0.33	1.60 ± 0.23	2.05 ± 0.32
3	0.89 ± 0.31	1.88 ± 0.91	1.78 ± 0.83	1.57 ± 0.50	1.05 ± 0.34
10	3.18 ± 0.61	2.81 ± 0.99	4.54 ± 1.23 ^a^	4.70 ± 0.76 ^a^	1.75 ± 0.57
30	11.77 ± 4.42	20.56 ± 4.83 ^a,d^	24.70 ± 7.15 ^a,d^	20.56 ± 3.93 ^a,d^	8.07 ± 1.47
**ACh (pmol)**					
1	−3.79 ± 1.00	−5.48 ± 0.63	−4.43 ± 0.87	−2.14 ± 0.30 ^a,b,c^	−4.18 ± 0.83
3	−6.07 ± 0.90	−5.95 ± 0.33	−6.03 ± 0.95	−3.14 ± 0.50 ^a,b,c,d^	−5.09 ± 0.51
10	−4.91 ± 0.52	−4.62 ± 0.44	−1.92 ± 0.83 ^a,c,d^	−2.17 ± 0.55 ^a,c,d^	−4.99 ± 0.55
30	−12.38 ± 1.22	−11.377 ± 1.45	−12.02 ± 1.18	−7.32 ± 0.50 ^a,b,c,d^	−10.19 ± 0.86
**SNP (pmol)**					
0.1	−5.06 ± 1.97	−3.61 ± 1.71	−5.04 ± 1.13	−2.96 ± 0.78	−3.89 ± 1.63
0.3	−4.40 ± 1.76	−3.50 ± 0.78	−4.48 ± 2.39	−5.02 ± 1.75	−4.05 ± 0.81
1	−5.36 ± 1.27	−4.58 ± 1.38	−5.15 ± 1.01	−4.90 ± 1.14	−4.39 ± 1.38
3	−4.40 ± 1.76	−7.18 ± 2.00	−5.80 ± 0.88	−6.35 ± 1.87	−6.82 ± 1.43

The data are expressed as the mean ± SEM of *n* = 8 rats per group compared with the control group (^a^ *p* < 0.05), LEAD group (^b^ *p* < 0.05), NOAD group (^c^ *p* < 0.05), and NEAD group (^d^ *p* < 0.05) using one-way ANOVA followed by Bonferroni’s post hoc test. Ach, acetylcholine; LEAD, normotensive and non-ovariectomized rats that received the atherogenic diet, methimazole, cholecalciferol, and l-NAME; LOAD, normotensive and ovariectomized rats that received the atherogenic diet, methimazole, cholecalciferol, and l-NAME; NEAD, normotensive and non-ovariectomized rats that received the atherogenic diet, methimazole, and cholecalciferol; NOAD, normotensive and ovariectomized rats that received the atherogenic diet, methimazole, and cholecalciferol; Phe, phenylephrine; SNP, sodium nitroprusside.

**Table 6:** Renal vascular reactivity in the various groups over 60 days of treatment. The data are presented as perfusion pressure variation (mmHg) after Phe, Ang II, ACh, and SNP administration.

Phe (nmol)	NEAD	NOAD	LEAD	LOAD	Control
0.1	1.39 ± 0.67	1.07 ± 0.68	1.84 ± 0.46	0.44 ± 0.55 ^a,c^	2.41 ± 0.58
0.3	2.32 ± 0.75	2.91 ± 0.99	1.90 ± 0.29	1.80 ± 0.48	2.49 ± 0.63
1	6.53 ± 2.51	8.00 ± 1.51	4.77 ± 0.99	2.02 ± 0.51 ^a,b,c,d^	6.96 ± 1.77
3	48.51 ± 10.71	85.10 ± 11.96	67.50 ± 6.51	56.85 ± 7.40	65.03 ± 12.83
**Ang II (pmol)**					
1	25.32 ± 3.91	29.31 ± 3.99	22.02 ± 3.66	15.33 ± 1.87 ^a,b,c,d^	23.76 ± 2.32
3	33.93 ± 5.89	45.00 ± 5.33	44.60 ± 9.43	48.22 ± 8.97	42.06 ± 10.49
10	33.43 ± 7.67	40.70 ± 7.99	47.39 ± 11.99	37.94 ± 5.84	39.44 ± 9.59
30	49.34 ± 8.16	64.70 ± 7.93	82.17 ± 13.08	68.55 ± 7.82	60.26 ± 8.54
**ACh (nmol)**					
0.03	−9.72 ± 2.26	−7.56 ± 2.90	−7.70 ± 1.58	−6.69 ± 1.54	−5.65 ± 1.52
0.1	−5.90 ± 1.12 ^a^	−5.48 ± 1.73 ^a^	−6.25 ± 1.80 ^a^	−6.67 ± 0.99 ^a^	−10.71 ± 0.66
0.3	−13.02 ± 2.23	−11.99 ± 1.83	−9.15 ± 2.99	−15.36 ± 2.71	−12.32 ± 1.84
1	−17.07 ± 4.10	−21.85 ± 2.98	−21.30 ± 2.37	−18.42 ± 2.15	−19.91 ± 2.43
**SNP (nmol)**					
0.3	−8.99 ± 1.67	−10.11 ± 1.20	−12.66 ± 2.54	−9.02 ± 0.99	−10.76 ± 2.19
1	−9.27 ± 1.12	−9.09 ± 1.69	−11.19 ± 1.91	−11.94 ± 1.94	−8.00 ± 2.39
3	−10.99 ± 1.55	−13.12 ± 1.23	−12.87 ± 1.98	−13.5 ± 1.12 ^a^	−14.36 ± 1.22
10	−12.66 ± 1.05	−15.78 ± 2.12	−14.51 ± 1.99	−14.24 ± 1.77	−15.95 ± 2.13

The data are expressed as the mean ± SEM of *n* = 8 rats per group compared with the control group (^a^ *p* < 0.05), LEAD group (^b^ *p* < 0.05), NOAD group (^c^ *p* < 0.05), and NEAD group (^d^ *p* < 0.05) using one-way ANOVA followed by Bonferroni’s post hoc test. Ach, acetylcholine; Ang II, angiotensin II; LEAD, normotensive and non-ovariectomized rats that received the atherogenic diet, methimazole, cholecalciferol, and l-NAME; LOAD, normotensive and ovariectomized rats that received the atherogenic diet, methimazole, cholecalciferol, and l-NAME; NEAD, normotensive and non-ovariectomized rats that received the atherogenic diet, methimazole, and cholecalciferol; NOAD, normotensive and ovariectomized rats that received the atherogenic diet, methimazole, and cholecalciferol; Phe, phenylephrine; SNP, sodium nitroprusside.

**Table 7:** Body weight and relative weight of the heart, kidney, and liver in the various groups over 60 days of treatment.

Parameter	NEAD	NOAD	LEAD	LOAD	Control
Initial body weight	237 ± 16.12	232 ± 10.1	240 ± 19.21	249 ± 17.12	241 ± 18.32
Final body weight	287 ± 20.21	299 ± 22.21	283 ± 19.21	300 ± 22.21	267 ± 19.91
Heart	0.28 ± 0.01	0.27 ± 0.01	0.25 ± 0.02	0.25 ± 0.02	0.26 ± 0.02
Kidney	0.33 ± 0.02	0.32 ± 0.02	0.29 ± 0.02	0.31 ± 0.01	0.31 ± 0.01
Liver	4.16 ± 0.15 ^a^	4.64 ± 0.30 ^a^	3.98 ± 0.20 ^a^	4.32 ± 0.27 ^a^	3.17 ± 0.15

The data are expressed as mean ± SEM of *n* = 8 rats per group compared with the control group (^a^ *p* < 0.05) using one-way ANOVA followed by Bonferroni’s post hoc test. LEAD, normotensive and non-ovariectomized rats that received the atherogenic diet, methimazole, cholecalciferol, and l-NAME; LOAD, normotensive and ovariectomized rats that received the atherogenic diet, methimazole, cholecalciferol, and L-NAME; NEAD, normotensive and non-ovariectomized rats that received the atherogenic diet, methimazole, and cholecalciferol; NOAD, normotensive and ovariectomized rats that received the atherogenic diet, methimazole, and cholecalciferol.

**Table 8:** Tissue redox status in the various groups over 60 days of treatment.

Heart	NEAD	NOAD	LEAD	LOAD	Control
SOD (mmol/hydroperoxides/mg protein)	27.92 ± 1.90	27.98 ± 1.83	34.09 ± 2.64	45.01 ± 3.07 ^a,c,d^	25.67 ± 1.18
CAT (µmol/min/mg protein)	272.8 ± 46.7	221.1 ± 32.62	325.2 ± 57.71	663.9 ± 77.59 ^a,b,c,d^	253.3 ± 42.96
LPO (U/mg protein)	202.7 ± 35.26	129.6 ± 22.95	134.9 ± 27.62	144.5 ± 39.82	164.7 ± 15.88
**Kidney**					
SOD (mmol/hydroperoxides/mg protein)	54.59 ± 6.38 ^a,b,c^	12.27 ± 1.63	14.79 ± 1.40	68.04 ± 14.43 ^a,b,c^	18.17 ± 4.54
CAT (µmol/min/mg protein)	1221.1 ± 153.1	695.4 ± 41.9 ^a,b,d,e^	1071.2 ± 125.4	1302.3 ± 92.8	1038 4 ± 116.0
LPO (U/mg protein)	262.9 ± 26.16 ^a,c^	83.44 ± 18.24	217.9 ± 23.93 ^a,c^	274.6 ± 41.7 ^a,c^	108.4 ± 7.52
**Aorta**					
SOD (mmol/hydroperoxides/mg protein)	22.41 ± 1.85	16.93 ± 1.34	24.65 ± 2.60	78.42 ± 3.19 ^a,b,c,d^	22.62 ± 0.81
CAT (µmol/min/mg protein)	618.3 ± 73.07 ^a,c^	275.6 ± 22.74	563.2 ± 78.26 ^a,c^	952.7 ± 72.55 ^a,b,c,d^	238.2 ± 14.35
LPO (U/mg protein)	204.62 ± 26.81 ^a,b^	44.25 ± 19.76 ^a,b,d^	128.21 ± 32.22	280.61 ± 35.39 ^a,b,c^	105.62 ± 9.31

The data are expressed as the mean ± SEM of *n* = 8 rats per group compared with the control group (^a^ *p* < 0.05), LEAD group (^b^ *p* < 0.05), NOAD group (^c^ *p* < 0.05), NEAD group (^d^ *p* < 0.05), and LOAD group (^e^ *p* < 0.05) using one-way ANOVA followed by Bonferroni’s post hoc test. CAT, catalase; LEAD, normotensive and non-ovariectomized rats that received the atherogenic diet, methimazole, cholecalciferol, and l-NAME; LOAD, normotensive and ovariectomized rats that received the atherogenic diet, methimazole, cholecalciferol, and l-NAME; LPO, lipoperoxides; NEAD, normotensive and non-ovariectomized rats that received the atherogenic diet, methimazole, and cholecalciferol; NOAD, normotensive and ovariectomized rats that received the atherogenic diet, methimazole, and cholecalciferol; SOD, superoxide dismutase.

**Table 9:** Cardiac and arterial morphometry in the various groups over 60 days of treatment.

Parameter (μm)	NEAD	NOAD	LEAD	LOAD	Control
Right ventricle	575.4 ± 87.12	688.5 ± 55.79	652.4 ± 18.71	666.4 ± 19.87	662.9 ± 77.23
Left ventricle	2113 ± 122.23	2399 ± 101.20	1845 ± 138.20	1925 ± 81.08	1755 ± 201.21
IV septum	6044 ± 703.11 ^a,b,c,e^	2103 ± 103.71	1749 ± 40.58	1434.1 ± 86.52	1309.2 ± 88.51
SA (intima layer)	64.80 ± 3.67 ^a,b,c,e^	3.10 ± 0.81	6.67 ± 0.81	4.54 ± 0.90	3.58 ± 0.57
CA (intima layer)	29.61 ± 2.49 ^a,b,c,e^	2.94 ± 0.27	3.68 ± 0.35	3.10 ± 0.46	2.94 ± 0.29

The data are expressed as the mean ± SEM of *n* = 8 rats per group compared with the control group (^a^ *p* < 0.05), LEAD group (^b^ *p* < 0.05), NOAD group (^c^ *p* < 0.05), and LOAD group (^e^ *p* < 0.05) using one-way ANOVA followed by Bonferroni’s post hoc test. CA, carotid artery; IV, interventricular; LEAD, normotensive and non-ovariectomized rats that received the atherogenic diet, methimazole, cholecalciferol, and l-NAME; LOAD, normotensive and ovariectomized rats that received the atherogenic diet, methimazole, cholecalciferol, and l-NAME; NEAD, normotensive and non-ovariectomized rats that received the atherogenic diet, methimazole, and cholecalciferol; NOAD, normotensive and ovariectomized rats that received the atherogenic diet, methimazole, and cholecalciferol; SA, subclavian artery.
